# Uva Ursi, Substitute for Ergot

**Published:** 1853-10

**Authors:** E. G. Harris


### *Uva Ursi, a substitute for Ergot.* By E. G. Harris, M. D.

I wish to call the attention of the medical profession to Uva Ursi, as a substitute for Ergot, in producing uterine contraction. Whether it will produce all the effects, or answer all the indications of that drug, I am not able to say; but I do know, that it will produce considerable uterine contraction when given during labor. Since December last, I have given it in five cases, in all of which it acted more efficiently than ergot. In three of these cases the pains had ceased entirely, from exaustion of nervous energy. A strong decoction of uva ursi was given every ten minutes, and in thirty minutes the pains had increased considerably, and in from one to one and a half hours delivery was effected and the placenta expelled, the uterus contracting well, and no untoward symptoms taking place. In the other two cases the pains had not ceased, but were fast doing so. I gave them bountifully to two of my *new parturients*—one of them was delivered in fifteen minutes, the placenta following in less than five; the other in an hour and twenty minutes, the placenta in ten. In all these cases the head occupied the superior strait at the time I commenced giving the medicine, in consequence, no doubt, of inefficient uterine contraction. The os uteri was in good condition in all the cases. Directly after the exhibition of the uva ursi, the pains would become strong and propulsive, lasting about the ordinary length of time; then going completely off, leaving the patient free from pain, except that produced by pressure upon the soft parts; the placenta soon followed; the uterus contracted well, and no hemorrhage more than ordinary. There was little or no tonic contraction until after the expulsion of the placenta, when it was complete.

I am aware these five cases are not sufficient to establish its reputation as a therapeutic agent in labor, yet the result of the past encourages me to gve it a further trial; and should its use in future be as successful in the hands of my professional brethren as it has in mine, I shall feel amply rewarded for any trouble I may have been at in bringing it to their notice. I was first induced to use it by being called to a case where the pains had ceased; and having no ergot, and knowing the effect it had on the kidneys and bladder, I gave it as above described. I prefer it to ergot for, the following reasons:

1st. Because there is no danger in it; you may give it *ad libitum.* It is well known that ergot often produces nausea and vomiting, and sometimes slow weak pulse, cold extremeties, dilation of the pupils, &c.

2d. Although it increases the propulsive efforts of the uterus, yet it does not produce that tonic contraction which is so painful to the mother, and at times so hazardous to the life of the child, until after the delivery is affected; this is generally far otherwise with ergot. More than once have I seen a young and healthy mother give birth to a well developed but dead infant—not from the poison being absorbed by the mother, and through the circulation destroying the child, but *alone* by the powerful tonic contraction of the uterus compressing the umbilical cord, arresting the circulation, &c. Often have I seen the placenta retained for hours, and in two cases for a day and night after it had been entirely detached from the uterns, by the firm and unyielding grasp of that organ—*all brought about by the free administration of ergot.*

My plan for giving is as follows:

℞. Uva ursa, a good article, 2 oz.
Boiling water, - - 1 pint.

Pour the water on the leaves in a pitcher or bowl, stir it till it becomes cool enough to drink, and give one fourth as hot as it can be drank every ten or fifteen minutes, until it has the desired effect. Attention should always be paid to the condition of the os uteri, dimensions of the pelvis, &c.—*Southern Med. and Surg. Jour.*

